# Predicting Body Height in a Pediatric Intensive Care Unit Using Ulnar Length

**DOI:** 10.3389/fped.2018.00187

**Published:** 2018-06-22

**Authors:** Melody A. Rasouli, Christopher J. L. Newth, Robinder G. Khemani, Patrick A. Ross

**Affiliations:** ^1^Keck School of Medicine, University of Southern California, Los Angeles, CA, United States; ^2^Children's Hospital Los Angeles, Keck School of Medicine of USC, University of Southern California, Los Angeles, CA, United States

**Keywords:** body height, ulnar length, anthropometry, intensive care unit, pediatrics

## Abstract

**Objective:** To determine if ulnar length obtained by the bedside nurse can be used to estimate patient length. To compare our findings to previous predictive equations of height and ulnar length. To evaluate the performance of predictive equations for height and ulnar length on patients with syndromes that affect height.

**Design:** Retrospective observational study of prospectively collected data.

**Settings:** Multidisciplinary Pediatric Intensive Care Unit in a university teaching hospital.

**Patients:** 1,177 patients, ages 1 month to 23 years. Mean age was 79.7 months (1,3 IQR 19.5, 164.5 months) and 55.4% male.

**Measurements:** Ulnar length was obtained using digital calipers by bedside nurses in PICU as well as height and weight. The electronic health care record was used to extract patient information.

**Main Results:** The predictive equation for height for the entire group is: height (cm) = 0.59^*^ulnar length (mm) + 13.1 (*r*^2^ = 0.93). Bland Altman analysis of the derivation formula applied to the testing group did not show any systematic bias.

**Conclusions:** Our study shows that ulnar length measurements can be used to predict height with a simple linear formula in a PICU setting. Not having specific individuals or specific training for ulnar measurement did not seem to alter the accuracy (*r*^2^ = 0.93). The robust nature of the measurement and ease of use may make this an unconventional but reasonable alternative to obtaining height when that cannot be measured directly.

## Introduction

Height is used in multiple calculations a pediatric intensive care unit (PICU) setting including: body mass index (BMI), body surface area, medication dosing (1), glomerular filtration (2) and prediction of pulmonary function (3). Unfortunately, height is not consistently recorded in many PICUs. Further, when height is measured in non-ambulatory patients, it is typically recumbent length which can underestimate height (4). The accuracy of measured height is also compromised by scoliosis, neuromuscular weakness, joint deformity, flexion contractures, and other syndromes (5, 6). Limb measurements such as arm span and ulnar length can be used to estimate height. It is known that among healthy children, predicting height using ulnar measurement is more accurate than measuring arm length (7–9) and there are reference values now from birth to 19.5 years. Studies demonstrated this by comparing measurements made by specifically trained researchers on healthy children. It is unknown whether the principle of using ulnar length to calculate height can be applied in a less controlled setting such as a PICU, on non-ambulatory children. This primary goal of this study is to determine if ulnar length obtained by the PICU bedside nurse can be used to estimate length. Secondary goals are to compare these results to previous predictive equations, to evaluate the performance of our equation for children with syndromes where accurate height may be difficult to obtain and to evaluate overall compliance with obtaining height measurements.

## Materials and methods

This observational study was conducted at a multidisciplinary PICU in a university teaching hospital over 2 years on patients ages 1 month to 23 years. The study was approved by the hospital's investigational review board with waiver of consent. We enrolled patients upon admission to the PICU and there were no criteria for exclusion. As part of a quality improvement project the nursing staff received a data sheet for values that also contained an illustration indicating how to measure ulnar length from the proximal end of the ulnar to the tip of the styloid process with the elbow bent between 90 and 110 degrees, as was explained by Gauld et al. (8). Nurses obtained weight using a combination of bedside scale, scaled lift or ICU bed with a scale system. Height is obtained by use of a paper tape stretched out alongside the recumbent patient. The bedside nurse obtained ulnar length using digital calipers. We used medical record numbers to extract further patient information including: chromosomal abnormality, genetic abnormality, inborn errors, scoliosis, thyroid disease, metabolic disorder, parathyroid disease, mitochondrial disorder, and multiple congenital defects. These syndromes were chosen to represent admission diagnosis which might alter measured height where ulnar length might be preserved.

Statistical analysis was performed using Statistica version 13 (Dell, Tulsa, OK). Linear regression of ulnar length and height was calculated for the entire group as well as those with and without identified syndromes. The correlation coefficient *r*^2^ and 95% confidence interval was generated. Data from non-syndromic patients was split evenly into derivation and testing groups. The derivation group was used to generate a predictive formula for height that was applied to the testing group. Previously published formulas from Gauld et al. (8) and von Ungern-Sternberg et al. (7) were also applied to the testing group. Patients with syndromes were evaluated with our predictive equation. Bland-Altman analysis was used to evaluate the presence of a systematic bias in application of the formula. The electronic health care record for the PICU was reviewed for the percentage of patients with recorded height.

## Results

**Figure 1:**
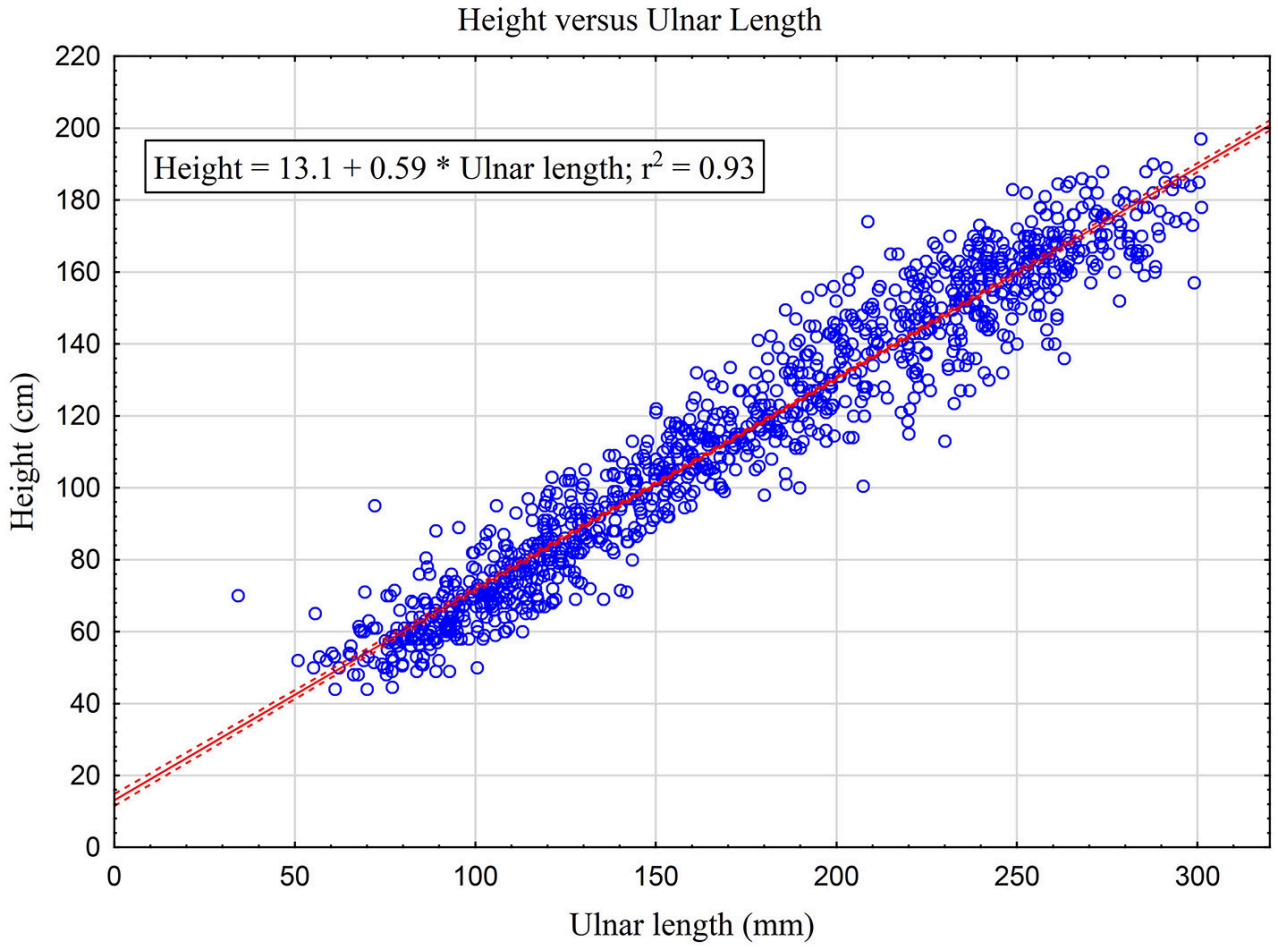
Relationship between height and ulnar length for all children in our study with linear regression prediction equation using a confidence interval of 95%.

A total of 1,216 patient episodes were collected. There was incomplete data in 39 leaving 1,177 children for analysis. There were 247 children identified that had at least one syndrome. The mean age was 79.7 months (1,3 IQR 19.5, 164.5 months) and the group was 55.4% male. The predictive equation for height for the entire group is: height (cm) = 0.59^*^ulnar length (mm) + 13.1 (*r*^2^ = 0.93; See Figure 1); for patients without syndromes: height (cm) = 0.59^*^ulnar length (mm) + 12.2 (*r*^2^ = 0.94); and for patients with syndromes: height (cm) = 0.56^*^ulnar length (mm) + 17.4 (r^2^ = 0.92). Data from the testing group was analyzed with formulas obtained from the derivation group, Gauld et al. (8) (5–19.5 years), and von Ungern-Sternberg et al. (7) (<5 years). The median bias and 95% confidence intervals of predicted minus measured length is presented in Table 1. Data from children with syndromes were analyzed in the same manner with the formula from the derivation group. Bland Altman analysis of the derivation formula applied to the testing group did not show any systematic bias (figure not shown). In the two years prior to the study the percentage of patients with height recorded was 40 and 67%. In the 2 years of the study the percentage was 84 and 87%.

**Table 1:** Mean bias and 95% limits of agreement for application of regression formulas.

	**Mean bias of predictive minus measured length**	**95% limits of agreement**
Children >5 years (*n* = 264) *Formula from derivation group*	−2.4 cm	−18.3 to 19.3 cm
Children >5 years (*n* = 264) *Formula from Gauld*	0.5 cm	−15.8 to 19.7 cm
Children <5 years (*n* = 201) *Formula from von Ungern-Sternberg*	−1.3 cm	−19.2 to 16.5 cm
Children with syndromes *Formula from derivation group*	0.6 cm	−19.0 to 20.1 cm

## Discussion

Our study shows that ulnar length measurements can be used to predict height in a PICU setting using values obtained by the bedside nurse. When the linear regression formula from the derivation group is applied to the testing group and compared against formulas from previous studies, it demonstrates that these formulas are similar in how they fit the data, and in that ulnar length predicted height. Our results are different in that a simple linear formula using just ulnar length was sufficient to identify this relationship. Other formulas require age and gender or natural log functions. This makes our formula fairly robust and can explain a good amount of the data. Further, it is unknown if formulas that have been developed in healthy populations can be applied directly to patients in an ICU setting. In one study by Prince et al. (10) the authors found that patients admitted to the PICU had a mean weight-for-age that was significantly lower than their UK reference mean. Even if formulas that are generated in healthy patients could be applied to PICU patients, the argument can be made to just measure height. Unfortunately, that is not often done as we realized reviewing compliance prior to the quality improvement study initiation. During the study the percentage of patients with height measured increased dramatically. We will re-evaluate in the future to see if this improvement is sustained and what proportion of height/length measurements are obtained by ulnar length compared to direct length measurement. The lack of height measurement can be pervasive in PICUs. In a retrospective study of US PICUs only 39.2% of 325,325 admissions had height recorded (11). Potentially, if we find a way to measure or estimate height more readily it may be measured more frequently. For recumbent patients measuring ulnar length is easier than measuring height. Further, we have some evidence that ulnar length may be used to predict ideal height in children that have syndromes. Many of our patients with significant neurodevelopmental problems are known to have contractures. We cannot get an accurate height and therefore cannot predict ideal weight. Using ulnar length avoids the issue of contractures.

There are several limitations to this study: We could not provide direct training in ulnar length measurement to every nurse. Nurses received instruction from other trained or experienced nurses as well as an informational handout. As patients were not measured by multiple nurses we cannot calculate inter or intra observer reliability of the technique. The patients were identified by a list of syndromes but we cannot be certain that all of those were affected by height. A large percentage of patients did not have ethnicity recorded in this group. We can report that our breakdown of race/ethnicity when our PICU submits information to national collaborative studies are: African American 8%, Asian/Indian/Pacific Islander 7%, Caucasian/European Non-Hispanic 18%, Hispanic 57%, other 8%, Unknown 2%. As patients were enrolled sequentially there is no reason to believe that the breakdown of race/ethnicity would be substantially different than our overall admissions. This in of its self may pose a limitation in that we do not yet know if our formula could be applied to PICUs with a different diversity. In spite of these limitations, measuring ulnar length may still be a robust way to obtain a surrogate for height. In our PICU there were 40 separate nurses who made the measurements and the training required was minimal. Not having specific individuals or specific training did not seem to alter the accuracy (*r*^2^ = 0.94). In turn, we believe that the technique could be easily applied to other PICUs and a formula defining the relationship between ulnar length and height could be generated for their population. With regards to future directions, further studies are needed to evaluate the accuracy of separate formulas for patients with syndromes such as scoliosis. We need to determine the inter-rater reliability and reproducibility of ulnar length measurement. We need to determine if it can be more accurate than recumbent length as compared to standing height. Finally, we would like to apply the use of ulnar length can be applied to other formulas to see if we can improve accuracy such as depth of endotracheal tube or esophageal probe insertion which we have previously presented in abstract form (12, 13).

## Conclusions

In conclusion, we feel that ulnar length can be used to predict height in a PICU population with minimal training for the staff. Given large potential for error in height or length measurements in critically ill patients, ulnar length appears to be an accurate and easily measureable surrogate. The robust nature of the measurement and ease of use may make this an unconventional but reasonable alternative to obtaining height when that cannot be measured directly.

## Ethics statement

The study was reviewed and approved by the Children's Hospital Los Angeles Investigational Review Board with waiver of informed consent.

## Author contributions

MR, PR conceptualized and designed the study; participated in data extraction; data analysis and interpretation of the data; participated in the drafting and revising the manuscript including final approval of the version to be published. CN, RK conceptualized and designed the study; participated in data analysis and interpretation of the data; participated in the drafting and revising the manuscript including final approval of the version to be published.

### Conflict of interest statement

The authors declare that the research was conducted in the absence of any commercial or financial relationships that could be construed as a potential conflict of interest.
